# Textile-supported silver nanoparticles as a highly efficient and recyclable heterogeneous catalyst for nitroaromatic reduction at room temperature†

**DOI:** 10.1039/c7ra13257c

**Published:** 2018-02-07

**Authors:** Wei Feng, Tingting Huang, Liqian Gao, Xianfeng Yang, Wenbin Deng, Rui Zhou, Hongjun Liu

**Affiliations:** Key Laboratory of Natural Medicine and Immuno-Engineering of Henan Province, Henan University Kaifeng Henan 475004 People's Republic of China 475004 hjliu@henu.edu.cn; School of Aerospace Engineering, Xiamen University 422 Siming South Road, Siming District Xiamen Fujian Province People's Republic of China 361005 rzhou2@xmu.edu.cn; School of Pharmaceutical Science (Shenzhen), Sun Yat-sen University Guang Dong Province People's Republic of China 510006; Analytical and Testing Center, South China University of Technology Guang Zhou People's Republic of China 510640

## Abstract

A novel textile-based nanosilver catalyst was prepared with a facile synthetic method. The textile-supported nanosilver (TsNS) proved to be an excellent heterogeneous catalyst for the reduction of nitroaromatics with a broad substrate scope. It can be recycled for up to 6 times without significantly compromising its catalytic efficacy. The TsNS catalyst was developed into a column reactor, demonstrating its practical application with the advantages of low cost, ease of operation and large scale synthesis capabilities. Scanning electron microscopy (SEM) showed that there were few changes to the catalyst's surface after the reaction. Besides, inductively coupled plasma (ICP) analysis showed that few silver particles leaked, and the interactions between the nitro groups of the nitroaromatics and the nanosilver particles were characterized by X-ray photoelectron spectroscopy (XPS), which lead to the proposal of a four-step mechanism for the reduction reaction.

## Abstract 2

A novel textile-supported nanosilver (TsNS) catalyst was prepared and applied in nitroaromatic reduction with excellent activity, stability and recyclability.

## Graphical abstract

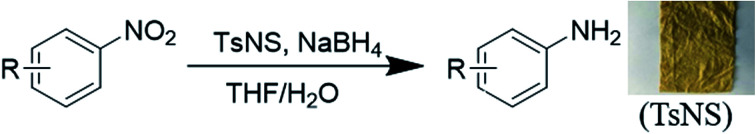

## Introduction

Environmental pollution is a global problem that is becoming increasingly serious with the fast worldwide economic growth. Nitroaromatics, such as nitro benzene and nitrophenols, are important and abundant organic pollutants in industry and agriculture.^1^ Among various techniques for the disposal of nitroaromatics,^2^ reduction of the nitro group into an amino group is desirable due to its high efficiency^3^ and the important applications of the reductive products in synthetic chemistry.^4^ Classic hydrogenation of nitro compounds employs various homogeneous transitional metal catalysts.^5–7^ Catalyst removal or recovery from the reaction mixture is one of the major challenges in green chemical processes and has hampered the industrialization of several novel catalytic systems.^8,9^ Heterogeneous catalysis is a desirable method to overcome this difficulty.^10–14^ A series of palladium nanoparticle catalysts supported on collagen fibres,^15^ carbon nanospheres,^16^ mesoporous silica^10^ and polymers,^17^ have been prepared and employed in the reduction of nitro compounds. Heterogeneous nanoparticle catalysts based on other metals, such as platinum,^18^ rhodium,^14^ gold^19^ and silver^3,20^ are also well developed. These catalysts are recycled either through filtration or *via* magnetic techniques. Although many heterogeneous catalysts presenting certain advantages, including reusability and cost effectiveness, have been developed, the economical preparation of highly reactive, scalable and recyclable heterogeneous catalysts is still a big challenge.

Recently, “textile catalysts” have appeared as a powerful approach for catalyst recovery and sustainable applications due to their low cost, ease of scaling and ease of operation.^21^ List has reported the preparation of an organo-textile catalyst by the formation of covalent bonds and its application in asymmetric synthesis with great enantioselectivity.^21^ Jiang has reported a wool-Pd-Co heterobimetallic catalyst for the asymmetric hydration of unsaturated carboxylic acids.^22^ We have been utilizing textile-supported silver nanoparticles as an antimicrobial material in health applications^23^ and water purification.^24^ Silver nanoparticles on textile will certainly be an advantage in terms of low cost, easy handling and endless supply of the supporting textile material. Such catalysts can be easily recycled by removal using a forceps and subsequent washing. Investigation of the interactions between chemicals and the nanosilver particles is also of great interest to understand the mechanism. As part of our continuous efforts towards textile-supported nanosilver (TsNS) applications^24,25^ and catalytic green chemistry,^26^ we herein report the first study on TsNS catalysts for nitroaromatic reduction.

## Experimental

### TsNS catalyst preparation

The TsNS catalyst was prepared following our previously reported procedure.^24^ Commercially available white cotton textile was washed with deionized water and dried in an oven at 60 °C. Silver nitrate (0.7 g) and (3-aminopropyl)triethoxysilane (APTES) (10 mL) were dissolved in deionized water (1 L). The dried cotton textile (30 g) was bathed in the solution at 60 °C for 10 min. The textile was then washed thoroughly with deionized water and soaked for 10 min in sodium borohydride (5 mM) solution at room temperature. A yellow layer appeared on the textile from the formation of silver nanoparticles on its surface. The textile catalyst was ready for use after rinsing with deionized water to remove excess reactants and un-bonded silver nanoparticles. It was then dried at room temperature in a drying cabinet. In line with our previously reported results, the weight percentage of silver in fabrics is 1.6%.

### General procedure for reduction of nitroaromatics

In a typical procedure, 0.2 mmol nitroaromatic compound, 1 mmol NaBH_4_ and 2 cm^2^ TsNS catalyst were mixed in THF/H_2_O (1 mL/1 mL) in a 20 mL glass vial and stirred at room temperature. The reaction was monitored by TLC until the starting material disappeared. Then, the reaction mixture was diluted with water and extracted with Et_2_O (5 mL × 3), the organic layers were combined and dried over Na_2_SO_4_, then concentrated and purified *via* column chromatography.

### Characterization

The samples were characterized by scanning electron microscopy (SEM; SEISS Merlin), Nuclear Magnetic Resonance imaging (NMR; 400 MHz Bruker Ultra Shield), X-ray Photoelectron Spectroscopy (XPS; PerkinElmer, PHI1600 spectrometer), and Inductively Coupled Plasma (ICP; Shimadzu ICPE-9800) analysis. The catalytic reactions were monitored by thin layer chromatography (TLC) purchased from Merck.

## Results and discussions

### TsNS catalysed reduction of nitroaromatics

**Fig. 1:**
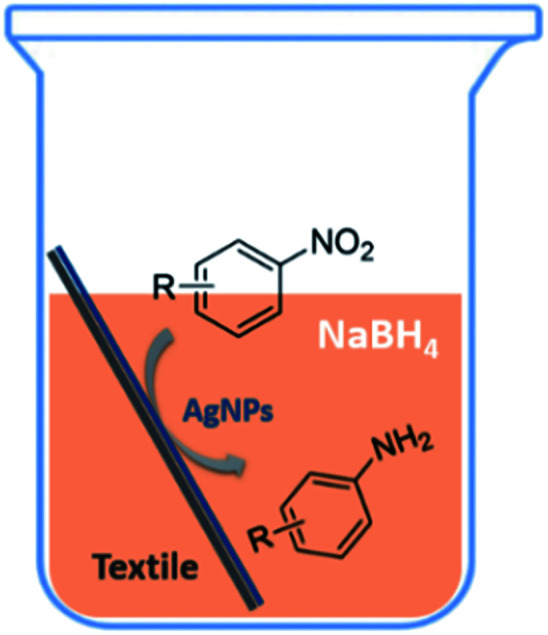
Schematic of the TsNS-catalysed reaction.

We tested the obtained TsNS catalyst in the reduction of nitroaromatics. As shown in Fig. 1, the nitro group was reduced into an amino group by NaBH_4_ with the TsNS catalyst. The products were confirmed by NMR and mass analysis. When 0.2 mmol 4-nitrophenol, 1 mmol NaBH_4_ and 2 cm^2^ TsNS catalyst were mixed in THF/H_2_O (1 mL/1 mL), TLC monitoring indicated the full consumption of the starting material within 3 hours at room temperature. No reaction was observed without the catalyst in a control experiment. When the same conditions were applied to a nitrile and an ester, such as 2-aminopyridine-4-carbonitrile and methyl benzoate or ethyl 2-phenyl acetate, no reaction occurred. The reaction scope was subsequently explored, and the results are summarized in Table 1.

**Table 1:** Scope of aryl nitro reduction reactions with the AgNPs/textile catalyst^a^

Entry	Substrate	Product	Time	Yield^b^
1	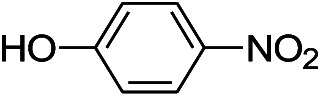	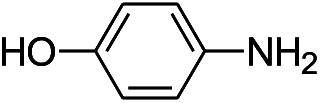	3 hours	73.4%
2	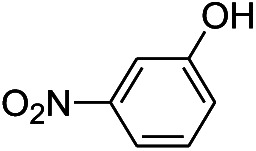	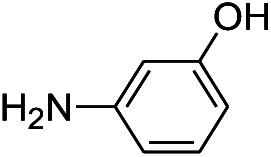	7 hours	54%
3^c^	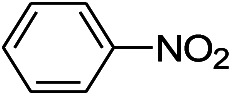	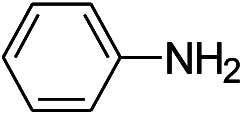	7 hours	81%
4	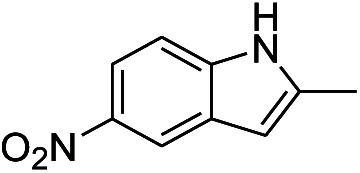	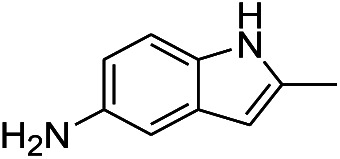	4 hours	70%
5	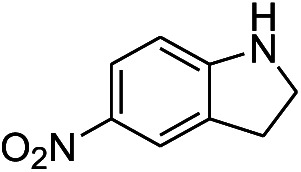	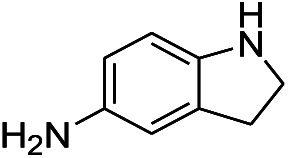	10 hours	91.2%
6	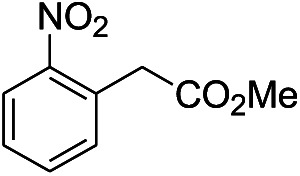	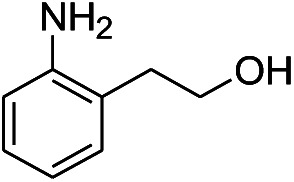	3 hours	55.6%
7	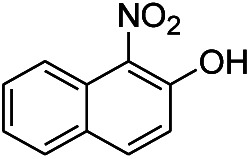	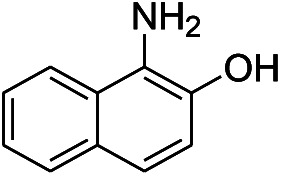	12 hours	33.5%
8	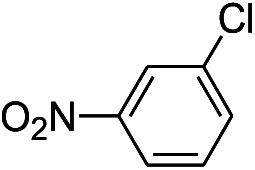	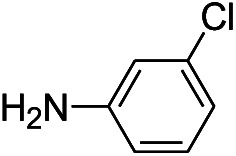	12 hours	58.3%
9	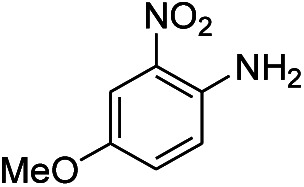	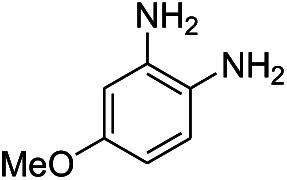	7 hours	72.5%

a Reaction conditions: 0.2 mmol substrate, 1 mmol NaBH_4_ and 2 cm^2^ TsNS catalyst were mixed in THF/H_2_O (1 mL/1 mL) in a 20 mL glass vial at room temperature.

b Isolated yield.

c Reaction carried out at 100 °C.

The reaction shows good tolerance of functional groups in nitroaromatics. 4-Nitrophenol (entry 1) and 3-nitrophenol (entry 2) with activated nitro groups are easily reduced at room temperature with moderate yields. Reduction of non-active nitroaromatics (entry 3) has to be carried out at a higher temperature. For 4-nitroindole (entry 4), the reduction can be finished in 4 hours with good isolated yield, while the reduction of nitro indoline is slightly slower and can only be finished in 10 hours with a yield of up to 91.2%. Even slower reduction was observed for 1-nitronaphthalen-2-ol (entry 7), which is not completely consumed even after 12 hours, and only 33.5% isolated yield can be achieved. It is surprising that when methyl 2-(2-nitrophenyl) acetate (entry 6) was applied to this condition, not only the nitro group, but also the ester group were reduced. The nitrogen atom probably plays the role of a directing group,^27^ which activated the ester group.

### Stability test for the catalyst

**Fig. 2:**
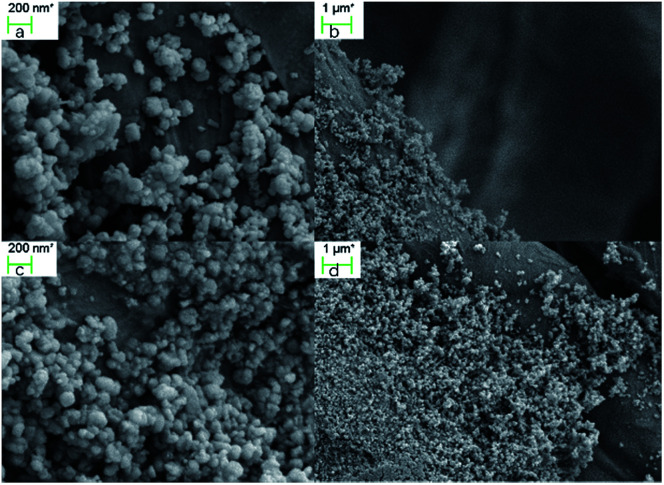
TsNS scan under FESEM before [a, b] and after [c, d] one catalytic process.

Stability is an important feature of a catalyst, and it has been evaluated by the amount of silver that leaked into the solution. Inductively coupled plasma (ICP) analysis of the reaction solution showed that only 60 ppb of silver was detected after the reaction completed, which is a very low level. The catalyst was also examined using field emission scanning electron microscopy (FESEM) before and after the catalytic process. As shown in Fig. 2, there are no significant changes in the silver loading and in its morphology in the textile before (a, b) and after (c, d) the reaction.

### Catalyst recycles

**Fig. 3:**
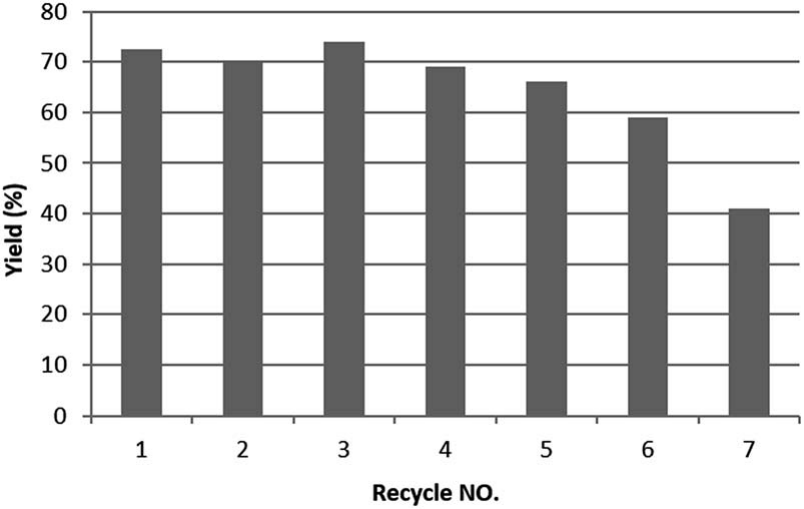
Catalyst recycling. 0.2 mmol substrate, 1 mmol NaBH_4_ and 2 cm^2^ TsNS catalyst were mixed in THF/H_2_O (1 mL/1 mL) in a 20 mL glass vial at room temperature. After 7 hours, the catalyst was removed and washed and used directly in the next round.

The low silver leakage from the TsNS catalyst into the reaction mixture provided a good opportunity to reuse the catalyst.^28^ Under the same condition as in Table 1, we tested the substrate 2-methyl-5-nitro-1*H*-indole (entry 4, Table 1) because of its good reactivity and ease of product separation by flash column (Fig. 3). The reaction time was extended to 7 hours, and in the first round, a similar isolated yield was achieved. The catalyst was then removed and washed with a THF/H_2_O mixture for direct use in the next round. The isolated yield only slightly decreased until the fifth round. The substrate was completely consumed for all rounds as indicated by TLC. However, the reaction could not be completed in round 6 and 7, and the isolated yield was only 59% and 41%, respectively.

### Large-scale catalytic reaction

Following above results, the catalyst was evaluated for its potential in larger scale reactions. 2-Methyl-5-nitro-1*H*-indole (entry 4, Table 1) was chosen as the model substrate due to its reactivity and significant difference between the NMR spectra of the substrate and the product. The results are summarized in Table 2. 1 to 5 mmol of substrate were tested with 2 cm^2^ TsNS catalyst loading. The reaction mixture was analysed by crude ^1^H NMR after 24 hours. Initially, the scale was increased by four, and at a scale of 1 mmol, the conversion reached 87%. However, it dropped significantly to 57.9% at a scale of 2 mmol. At scales of 3, 4, 5 mmol, the conversion further dropped to 42.9%, 40.1% and 33.3%, respectively.

**Table 2:** Screening of the reaction scale^a^

Entry	Substrate amount	THF volume	H_2_O volume	Conversion^b^
1	1 mmol	1 mL	1 mL	87.0%
2	2 mmol	2 mL	2 mL	57.9%
3	3 mmol	3 mL	3 mL	42.9%
4	4 mmol	4 mL	4 mL	40.1%
5	5 mmol	5 mL	5 mL	33.3%

a Reaction conditions: 1 equivalent of substrate, 5 equivalents of NaBH_4_ and 2 cm^2^ TsNS catalyst were mixed in THF/H_2_O in a 20 mL glass vial at room temperature.

b Determined by ^1^H NMR after 4 hours.

### Flow chemistry

**Fig. 4:**
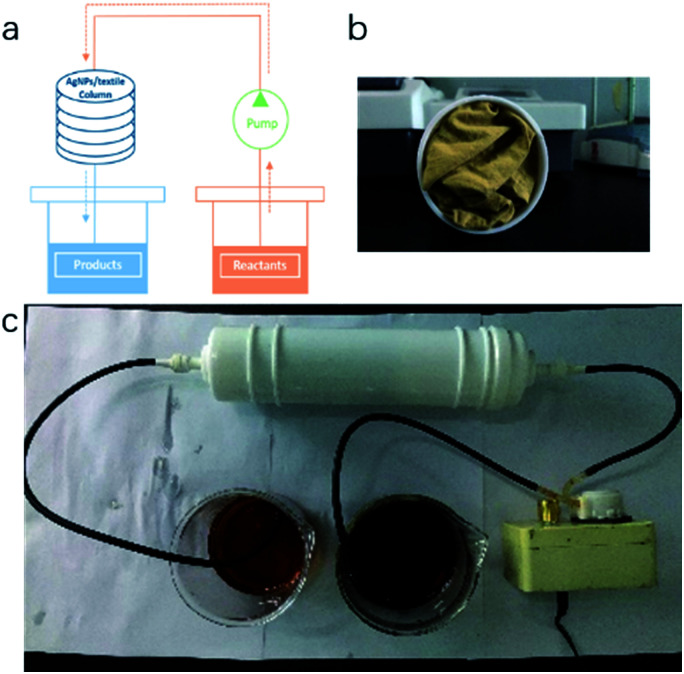
Schematic of TsNS catalysis in a flow chemistry system. [a] Flow system, [b] inside of the column, [c] picture of the flow system.

Our TsNS catalyst was then tightly packed into a column (diameter of 4 cm) and tested for its potential application in flow chemistry. 3.4 g (20 mmol) 4-methoxy-2-nitroaniline (entry 9, Table 1) and 4 g NaBH_4_ were dissolved in a mixture of THF/H_2_O (200 mL/200 mL). The solution was pressured to flow slowly through the column within 4 hours, as shown in Fig. 4. After that, the column was flushed with a THF and water mixture (1/1 ratio in volume) to ensure that no reactants or products remained inside the column. The conversion was determined by crude ^1^H NMR to be 80%. In the second test, using the same column and the same amount of starting material, similar conversion was achieved, which indicated the good stability and high catalytic efficiency of our catalyst in the application of flow chemistry.

### Mechanism study

**Fig. 5:**
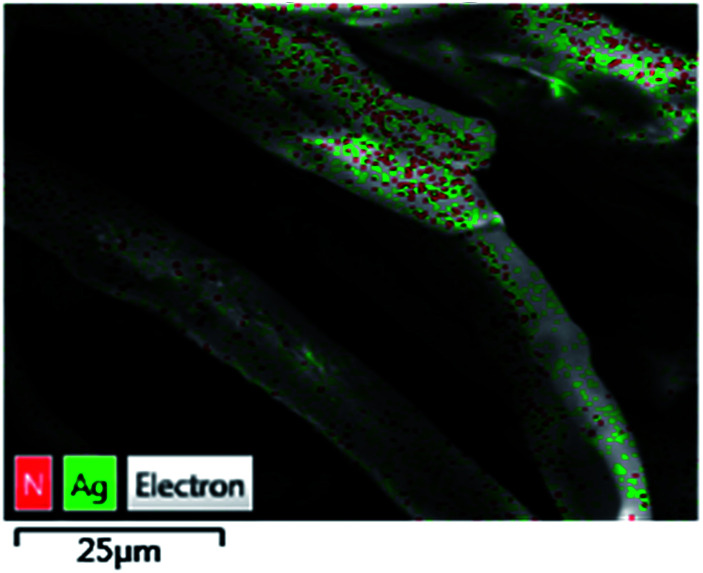
EDX-mapping of the TsNS catalyst.

The TsNs was soaked in nitrobenzene (entry 3, Table 1) for 7 hours to study the interactions between the nitrobenzene and silver nanoparticles in the absence of NaBH_4_. The textile was then dried and analysed by the EDX-mapping of Ag and N. As shown in Fig. 5, Ag spreads evenly on the fibres. Meanwhile, N element spreads evenly over Ag, indicating possible interactions between Ag and the nitro group of nitrobenzene.

**Fig. 6:**
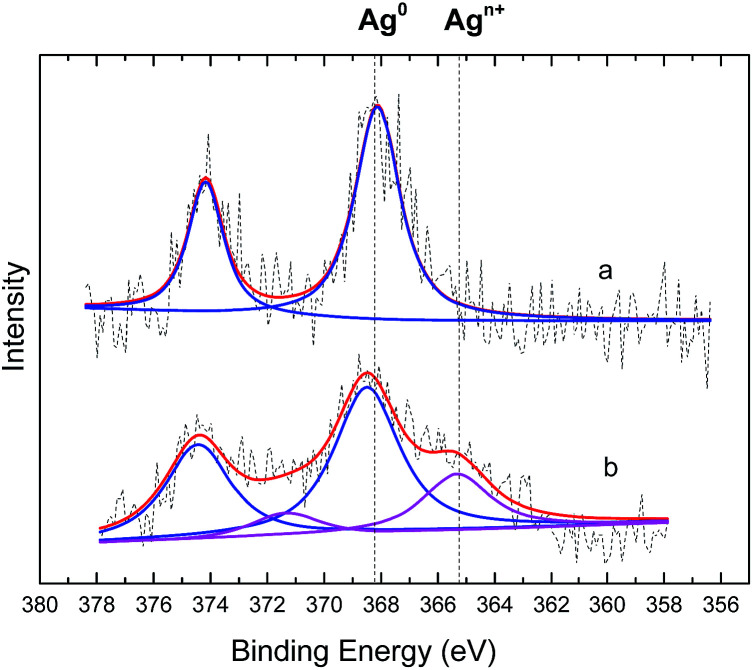
XPS study of the TsNS catalyst. (a) Non-treated TsNS (b) nitrobenzene-treated TsNS.

Nitrobenzene-treated TsNS and non-treated TsNS were subjected to X-ray photoelectron spectroscopy (XPS) measurements to further understand the mechanism. The XPS spectra were corrected by C 1s. As shown in Fig. 6, the XPS peaks of the non-treated TsNS (Fig. 6a) for the Ag 3d_3/2_ and Ag 3d_5/2_ signals appeared at a binding energy of 374.3 eV and 368.3 eV, respectively, which indicated the metallic nature of Ag(0) on the textile.^29^

In contrast, the XPS spectra of nitrobenzene-treated TsNS contained two pairs of peaks (Fig. 6b). The major pair at 374.5 and 368.5 eV corresponded to Ag^0^. The minor one showed an energy shift of 2.4 eV, resulting in a binding energy of 372.1 eV and 366.1 eV for Ag 3d_3/2_ and Ag 3d_5/2_, respectively. In addition, the full width at half maximum (FWHM) of Ag^0^ is 2.24 eV (ref. 30), while the shift spectrum has a broader FWHM of 2.79 eV.

It is known that Ag(ii)O^31^ has a binding energy of 373.3 eV and 367.3 eV for Ag 3d_3/2_ and Ag 3d_5/2_, respectively. Ag(i)_2_O (ref. 32) has a binding energy of 373.7 eV and 367.7 eV for Ag 3d_3/2_ and Ag 3d_5/2_, respectively. The shift between Ag and Ag oxide is as small as 1.0 eV, which is much smaller than the shift of 2.4 eV that was observed in our analysis. This is very likely due to some other oxidized forms of silver, which are probably formed through coordination between the silver nanoparticles in the TsNS and the oxygen atoms in the nitro groups of nitrobenzene. All the above results demonstrated that Ag(0) is the active form in our TsNS catalyst and that it is oxidized through coordination with nitro groups, thus activating the substrate.

**Fig. 7:**
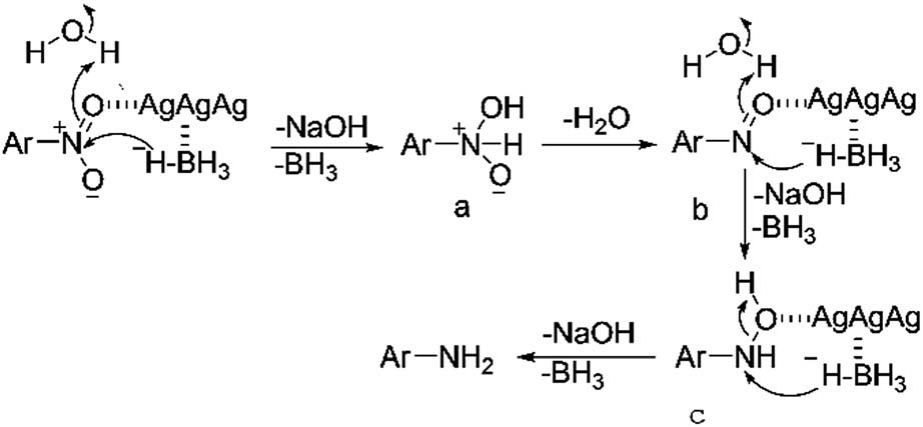
Proposed mechanism.

Supported by the above analysis, the TsNS catalysis in this reaction might work through a Langmuir–Hinshelwood mechanism,^33^ which is proposed in Fig. 7. The silver nanoparticles could absorb the nitroaromatics and hydrides onto its surface, which increased the reduction possibility. With the nitroaromatics activated by silver on the surface of the textile catalyst, the hydride can attack the nitrogen atom to deliver intermediate a, which quickly loses one molecule of water to generate nitroso b. Repetition of the reduction process will successively produce hydroxylamine c and the final product. This proposal is supported by Pal's work on the kinetics of aryl nitro reduction with NaBH_4_.^34^

## Conclusions

In conclusion, silver nanoparticles were successfully immobilized onto a cotton textile by a facile synthetic process. This TsNS catalyst has been proven to be efficient in the reduction of nitroaromatics. It can be recycled up to 6 times without significantly decreasing its catalytic efficacy. When applied in flow chemistry, this catalyst showed high conversion efficiency of nitroaromatic pollutants to useful amine products on a relatively large scale. In addition, the catalyst showed good stability, which was confirmed by ICP analysis. SEM images showed no obvious changes on the surface of the catalyst before and after reactions. By using EDX-mapping and XPS analysis, a strong coordination between the Ag and nitro groups was observed, which provided good explanation for its high activity and led to a deep understanding of the catalytic mechanism. Further research on the potential applications of this TsNS catalysis in environmental pollution treatment is in progress.

## Conflicts of interest

There are no conflicts to declare.

## Supplementary Material

RA-008-C7RA13257C-s001

## References

[cit1] Chang Y. C., Chen D. H. (2009). J. Hazard. Mater..

[cit2] O'Connor O. A., Young L. Y. (1989). Environ. Toxicol. Chem..

[cit3] Muniz-Miranda M. (2014). Appl. Catal., B.

[cit4] Zhang Y., Yuan X., Wang Y., Chen Y. (2012). J. Mater. Chem..

[cit5] Rahaim Jr R. J., Maleczka Jr R. E. (2005). Org. Lett..

[cit6] Shil A. K., Das P. (2014). Green Chem..

[cit7] Schabel T., Belger C., Plietker B. (2013). Org. Lett..

[cit8] O'Neal E. J., Lee C. H., Brathwaite J., Jensen K. F. (2015). ACS Catal..

[cit9] Battilocchio C., Iannucci G., Wang S., Godineau E., Kolleth A., De Mesmaeker A., Ley S. (2017). React. Chem. Eng..

[cit10] Kim A., Rafiaei S. M., Abolhosseini S., Shokouhimehr M. (2015). Energy & Environment Focus.

[cit11] Shokouhimehr M. (2015). Catalysts.

[cit12] Janssen M., Mueller C., Vogt D. (2011). Cheminform.

[cit13] Yang X. F., Wang A., Qiao B., Li J., Liu J., Zhang T. (2013). Acc. Chem. Res..

[cit14] Luo P., Xu K., Zhang R., Huang L., Wang J., Xing W., Huang J. (2012). Cheminform.

[cit15] Wu H., Zhuo L., He Q., Liao X., Shi B. (2009). Appl. Catal., A.

[cit16] Lu Y. M., Zhu H. Z., Li W. G., Hu B., Yu S. H. (2013). J. Mater. Chem. A.

[cit17] Johnson J. A., Makis J. J., Marvin K. A., Rodenbusch S. E., Stevenson K. J. (2013). J. Phys. Chem. C.

[cit18] Lara P., Philippot K. (2015). Catal. Sci. Technol..

[cit19] Kuroda K., Ishida T., Haruta M. (2009). J. Mol. Catal. A: Chem..

[cit20] Liu J., Cui J., Vilela F., He J., Zeller M., Hunter A. D., Xu Z. (2015). Chem. Commun..

[cit21] Lee J. W., Mayer-Gall T., Opwis K., Song C. E., Gutmann J. S., List B. (2013). Science.

[cit22] Wang S.-Q., Wang Z.-W., Yang L.-C., Dong J.-l., Chi C.-Q., Sui D.-N., Wang Y.-Z., Ren J.-G., Hung M.-Y., Jiang Y.-Y. (2007). J. Mol. Catal. A: Chem..

[cit23] Kim S. S., Park J. E., Lee J. (2011). J. Appl. Polym. Sci..

[cit24] Liu H., Tang X., Liu Q. (2014). J. Water Health.

[cit25] Liu H., Lee Y.-Y., Norsten T. B., Chong K. (2013). J. Ind. Text..

[cit26] Liu H., Feng W., Kee C. W., Zhao Y., Leow D., Pan Y., Tan C.-H. (2010). Green Chem..

[cit27] Leow D., Li G., Mei T. S., Yu J. Q. (2012). Nature.

[cit28] Mizuno N., Misono M. (1998). Chem. Rev..

[cit29] Jussila P., Lahtonen K., Lampimäki M., Hirsimäki M., Honkanen M., Lepistö T., Taskinen P., Valden M. (2008). Surf. Sci. Spectra.

[cit30] Politano A., Chiarello G. (2009). Surf. Rev. Lett..

[cit31] Hoflund G. B., Weaver J. F., Epling W. S. (1994). Surf. Sci. Spectra.

[cit32] Hoflund G. B., Weaver J. F., Epling W. S. (1994). Surf. Sci. Spectra.

[cit33] Wang M., Tian D., Tian P., Yuan L. (2013). Appl. Surf. Sci..

[cit34] Aditya T., Pal A., Pal T. (2015). Chem. Commun..

