# Supplementary material for: Textile-supported silver nanoparticles as a highly efficient and recyclable heterogeneous catalyst for nitroaromatic reduction at room temperature
Source: RSC Adv. 2018 Feb 7;8(12):6288–92. doi: 10.1039/c7ra13257c (PMC9078239; doi:10.1039/c7ra13257c)
Supplement: RA-008-C7RA13257C-s001 [file RA-008-C7RA13257C-s001.pdf]

Electronic Supplementary Material (ESI) for RSC Advances.
This journal is © The Royal Society of Chemistry 2018

Supporting Information

Textile supported silver nanoparticles as highly efficient and recyclable heterogeneous catalyst for nitroaromatics reduction at room temperature

Wei Feng,[c] Tingting Huang,[b] Liqian Gao,[c] Xianfeng Yang,[d] Wenbin Deng,[c] Rui Zhou[b,*] and Hongjun Liu[a,*]

a Key Laboratory of Natural Medicine and Immuno-Engineering of Henan Province, Henan University, Kaifeng, Henan 475004, People's Republic of China 475004

b School of Aerospace Engineering, Xiamen University, 422 Siming South Road, Siming District, Xiamen, Fujian Province, People's Republic of China 361005

c School of Pharmaceutical Sciences, Sun Yat-sen University Shenzhen, Guang Dong Province, People's Republic of China 510006

d Analytical and Testing Center, South China University of Technology, Guang Zhou, People's Republic of China, 510640

a* email: hjliu@henu.edu.cn

b* email: rzhou2@xmu.edu.cn

Contents

1 General Procedures and Methods

2 Representative procedure for nanotextile catalyst preparation

3 Reaction optimization table

4 Representative procedure for nanotextile catalyzed nitroaromatic reduction

5 NMR and mass data for products

6 NMR Spectra

1 General Procedures and Methods

Proton nuclear magnetic resonance (1H NMR) and carbon NMR (13C NMR) spectra were recorded in CD_3OD and $CDCl_3$ unless otherwise stated. 1H (400 MHz) and 13C (101 MHz) with complete proton decoupling were performed on a 400 MHz Bruker Ultra Shield NMR spectrometer. Chemical shifts were reported as δ in units of parts per million (ppm) downfield from tetramethylsilane (δ 0.00), using the residual solvent signal as an internal standard: CD_3OD (1H NMR, δ 3.31; 13C NMR, δ 49.0), $CDCl_3$ (1H NMR, δ 7.26 singlet; 13C NMR, δ 77.0 triplet). Multiplicities were given as s (singlet), d (doublet), t (triplet), q (quartet), m (multiplet), dd (doublet of doublets), ddd (doublet of doublet of doublets), dt (doublet of triplets), dq (doublet of quartets), and br (broad). Coupling constants (J) were recorded in hertz (Hz). The number of proton atoms (n) for a given resonance was indicated by nH. MS was reported in units of mass to charge ratio (m/z). Mass samples were dissolved in MeCN (AR grade). X-Ray Photoelectron Spectroscopy (XPS) was performed on a PerkinElmer, PHI1600 spectrometer. Inductively Coupled Plasma (ICP) was carried out on a Shimadzu ICPE-9800 Series system.

All commercial reagents were purchased from Sigma- Aldrich, Fluka, Alfa Aesar, Merck, TCI, or Acros and were of the highest purity grade available. They were used without further purification unless specified. Reaction solvents were AR grade THF and deionized water.

2 Representative procedure for textile supported nanosilver catalyst preparation

Commercially available white cotton textile was washed with deionized water and dried in oven at 60 °C. Silver nitrate (0.7 g) and (3-Aminopropyl) triethoxysilane (APTES) (10ml) were dissolved in deionized water (1L). The dried cotton textile (30g) was bathed in the solution at 60 °C for 10 min. The textile was then washed thoroughly with deionized water and soaked for 10 min in a sodium borohydride (5 mM) solution at room temperature. A yellow color was formed on the textile due to the formation of silver nanoparticles on its surface. The textile was ready to use after rinsing with deionized water to remove any excess reactants and un-bonded silver nanoparticles. It was then dried at room temperature in drying cabinet.

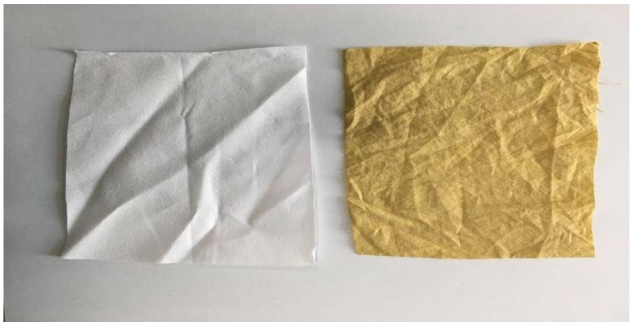

Figure S1, Picture of textile before (white color) and after (yellow color) coating with silver nanoparticles.

3 Reaction optimizations

Table S1 Optimization of TsNS catalyzed 4-nitrophenol reduction.

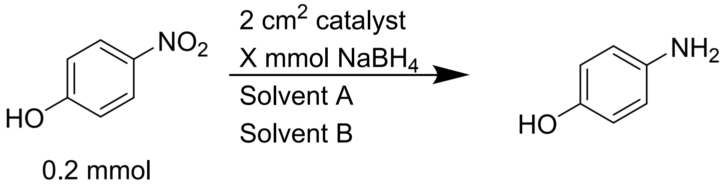

Entry	Solvent A	Solvent B	X	Time (hours)	Yield (%)[a]
**1[b]**	**2 ml DCM**		**1**	**12**	**trace**
**2 [b]**	**2 ml MeCN**		**1**	**12**	**~8%**
**3 [b]**	**2ml THF**		**1**	**12**	**~10%**
**4**	**1ml THF**	**1ml H_2O**	**1**	**3**	**73%**
**5**	**1ml THF**	**1ml H_2O**	**0.5**	**3**	**46%**
**6**	**1ml THF**	**1ml H_2O**	**2**	**3**	**75%**
**7 [b]**	**1.5 ml THF**	**0.5 ml H_2O**	**1**	**3**	**50%**
**8 [c]**	**0.5**	**1.5**	**1**	**3**	**26%**

Nitrophenol, catalyst and $NaBH_4$ were mixed in solvent and stirred at room temperature. [a] isolated yield. [b] $NaBH_4$ is not completely dissolved. [c] nitrophenol is not completely dissolved. Entry 4 is the best condition.

4 Representative procedure for nanotextile catalyzed nitroaromatic reduction

To a 25ml rbf was added a magnetic stirring bar, 2 cm^2 nanosilver textile catalyst, 0.2 mmol nitro compound and 1mmol $NaBH_4$, following this, 1ml THF and 1ml deionized water were added. The mixture was stirred at room temperature (25 ^oC, 100 ^oC for certain substrate) until substrate was completely consume as indicated by TLC monitoring. Then the catalyst was taken out and washed thoroughly with deionized water, which was combined with the reaction mixture. Extract with Ether for three times and the organic layers were combined and dried with Na_2SO_4, concentrated and purified by flash chromatography.

5 NMR and Mass data for products

All compounds were purified by flashing silica chromatography with hexane and ethyl acetate (EA) mixture as eluent.

Compound 1: 4-aminophenol

^{1}H NMR (400 MHz, MeOD) δ 6.62 (td, J = 9.0, 6.4 Hz, 4H). ^{13}C NMR (101 MHz, MeOD) δ 151.32, 140.22, 118.54, 116.74. LRMS (ESI) m/z 110.0 (M + H^+). Eluent: Hexane/ EA = 5/1.

Compound 2: 2-methyl-1H-indol-5-amine

^{1}H NMR (400 MHz, MeOD) δ 7.05 (d, J = 8.4 Hz, 1H), 6.83 (d, J = 2.0 Hz, 1H), 6.57 (dd, J = 8.4, 2.1 Hz, 1H), 5.92 (s, 1H), 2.35 (s, 3H). ^{13}C NMR (101 MHz, MeOD) δ 139.20, 137.04, 133.32, 131.34, 112.81, 111.55, 107.03, 99.49, 13.49. LRMS (ESI) m/z 147.2 (M + H^+). Eluent: Hexane/ EA = 1/1.

Compound 3: indolin-5-amine

^{1}H NMR (400 MHz, $CDCl_3$) δ 6.61 – 6.56 (m, 1H), 6.52 (d, J = 8.1 Hz, 1H), 6.42 (dd, J = 8.1, 2.3 Hz, 1H), 3.49 (t, J = 8.3 Hz, 2H), 3.16 (br, 3H), 2.95 (t, J = 8.2 Hz, 2H). ^{13}C NMR (101 MHz, $CDCl_3$) δ 144.11, 139.15, 131.27, 114.27, 113.15, 110.76, 47.57, 30.47. LRMS (ESI) m/z 135.0 (M + H^+). Eluent: Hexane/ EA = 1/1.

Compound 4: aniline

^{1}H NMR (400 MHz, MeOD) δ 7.24 – 7.14 (m, 2H), 7.00 – 6.93 (m, 2H), 6.85 (tt, J = 7.5, 1.1 Hz, 1H). ^{13}C NMR (101 MHz, MeOD) δ 152.69, 129.61, 121.97, 115.14. LRMS (ESI) m/z 93.9 (M + H^+). Eluent: Hexane/ EA = 10/1.

Compound 5: 2-(2-aminophenyl) ethanol

^{1}H NMR (400 MHz, $CDCl_3$) δ 7.07 (t, *J* = 8.2 Hz, 2H), 6.85 – 6.61 (m, 2H), 3.90 (t, *J* = 6.1 Hz, 2H), 2.96 (br, 3H), 2.80 (t, *J* = 6.1 Hz, 2H). ^{13}C NMR (101 MHz, $CDCl_3$) δ 144.81, 130.60, 127.69, 124.40, 119.37, 116.39, 63.32, 34.75. LRMS (ESI) m/z 138.1 (M + H^+). Eluent: Hexane/ EA = 1/1.

Compound 6: 3-aminophenol

^{1}H NMR (400 MHz, MeOD) δ 7.70 (ddd, *J* = 8.2, 2.2, 0.9 Hz, 1H), 7.62 (t, *J* = 2.3 Hz, 1H), 7.42 (t, *J* = 8.2 Hz, 1H), 7.18 (ddd, *J* = 8.2, 2.4, 0.9 Hz, 1H). ^{13}C NMR (101 MHz, MeOD) δ 159.81, 150.65, 131.26, 122.92, 115.09, 110.87. LRMS (ESI) m/z 110.0 (M + H^+). Eluent: Hexane/ EA = 3/1.

Compound 7: 1-aminonaphthalen-2-ol

^{1}H NMR (400 MHz, MeOD) δ 7.86 (dd, *J* = 8.5, 0.7 Hz, 1H), 7.68 (d, *J* = 8.2 Hz, 1H), 7.36 (ddd, *J* = 8.4, 6.8, 1.3 Hz, 1H), 7.23 (ddd, *J* = 8.8, 5.5, 2.1 Hz, 2H), 7.08 (d, *J* = 8.7 Hz, 1H). ^{13}C NMR (101 MHz, MeOD) δ 141.79, 130.50, 129.16, 128.04, 126.61, 125.79, 123.68, 121.58, 120.14, 118.31. LRMS (ESI) m/z 182.3 (M + Na^+). Eluent: Hexane/ EA = 1/1.

Compound 8: 3-chloroaniline

^{1}H NMR (400 MHz, MeOD) δ 7.14 (t, *J* = 8.0 Hz, 1H), 6.96 (t, *J* = 2.1 Hz, 1H), 6.84 – 6.77 (m, 2H). ^{13}C NMR (101 MHz, MeOD) δ 154.53, 135.54, 130.83, 121.12, 114.46, 112.90. LRMS (ESI) m/z 127.9 (M + H^+). Eluent: Hexane/ EA = 10/1.

Compound 9: 4-methoxybenzene-1,2-diamine

^{1}H NMR (400 MHz, $CDCl_3$) δ 6.63 (d, *J* = 8.4 Hz, 1H), 6.31 (d, *J* = 2.7 Hz, 1H), 6.25 (dd, *J* = 8.3, 2.7 Hz, 1H), 3.72 (s, 3H), 3.29 (s, 4H). ^{13}C NMR (101 MHz, $CDCl_3$) δ 154.60, 137.09, 127.36, 118.34, 104.10, 102.96, 55.54. LRMS (ESI) m/z 139.0 (M + H^+). Eluent: Hexane/ EA = 3/1.

6 NMR Spectra

Compound 1 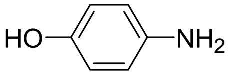

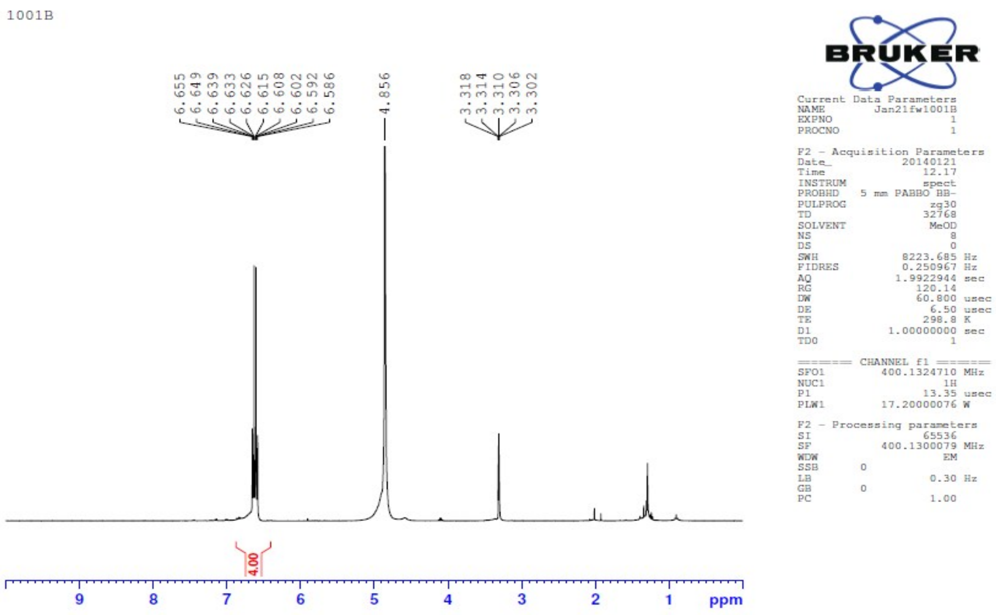

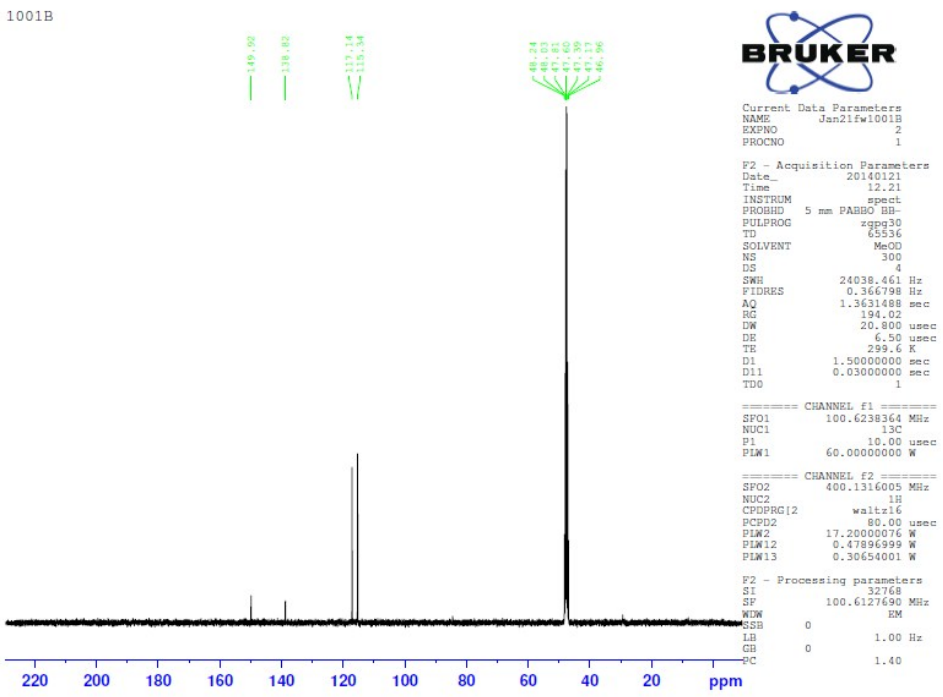

Compound 2

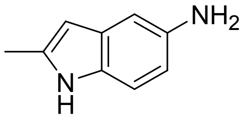

NH2
N
H

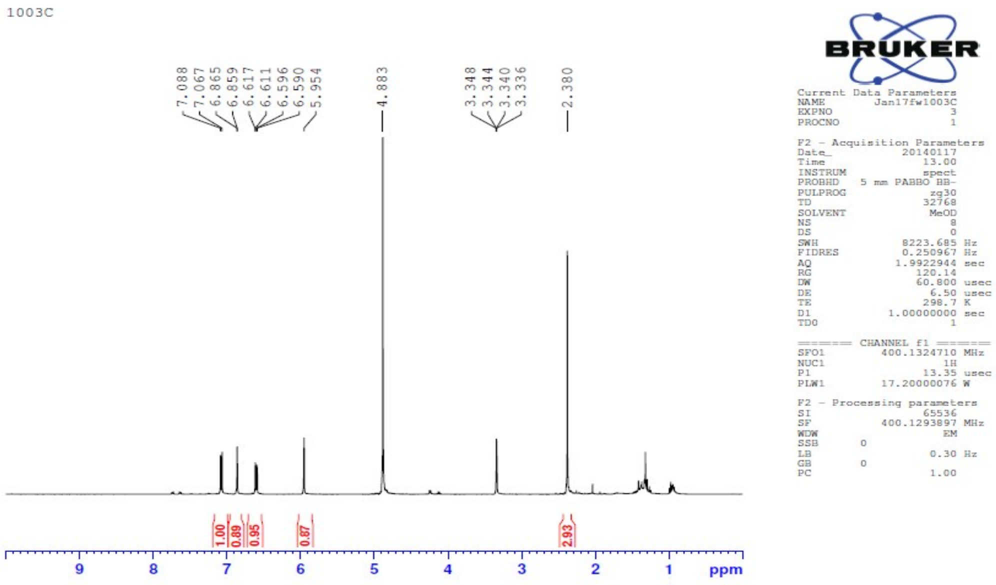

1003C
7.088
7.067
6.865
6.859
6.617
6.611
6.596
6.590
5.954
4.883
3.348
3.344
3.340
3.336
2.380
1.00
0.89
0.95
0.87
2.93
9
8
7
6
5
4
3
2
1
ppm
BRUKER
Current Data Parameters
NAME Jan17fw1003C
EXPNO 3
PROCNO 1
F2 - Acquisition Parameters
Date_ 20140117
Time 13.00
INSTRUM spect
PROBHD 5 mm PABBO BB-
PULPROG zg30
TD 32768
SOLVENT MeOD
NS 8
DS 0
SWH 8223.685 Hz
FIDRES 0.250967 Hz
AQ 1.9922944 sec
RG 120.14
DW 60.800 usec
DE 6.50 usec
TE 298.7 K
D1 1.00000000 sec
TD0 1
======= CHANNEL f1 =======
SFO1 400.1324710 MHz
NUC1 1H
P1 13.35 usec
PLW1 17.20000076 W
F2 - Processing parameters
SI 65536
SF 400.1293897 MHz
WDW EM
SSB 0
LB 0.30 Hz
GB 0
PC 1.00

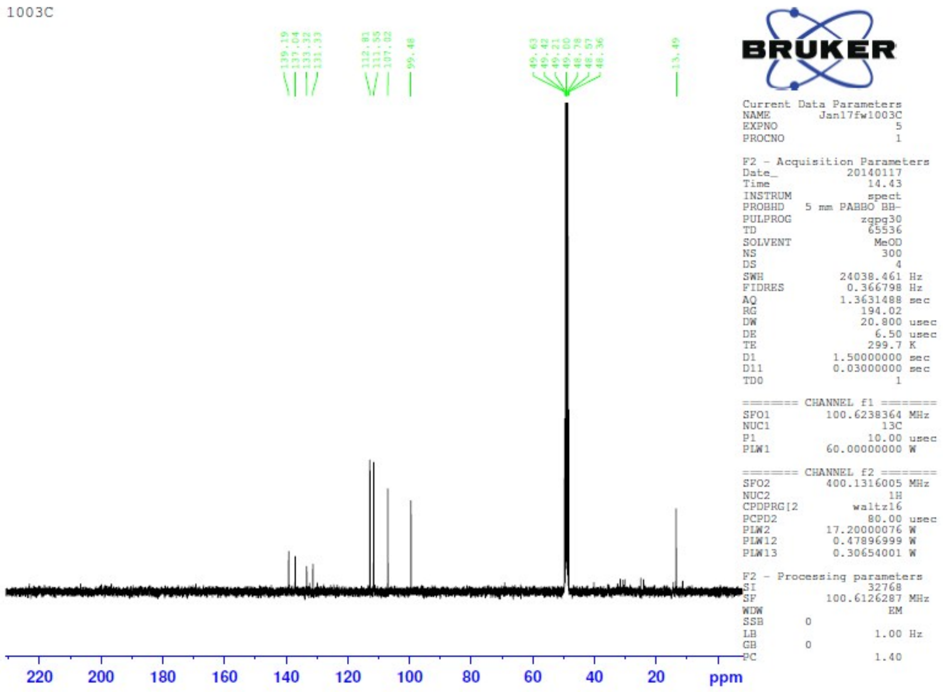

1003C
139.19
137.04
133.32
131.33
112.81
111.55
107.02
99.48
49.63
49.42
49.21
49.00
48.78
48.57
48.36
13.49
220
200
180
160
140
120
100
80
60
40
20
ppm
BRUKER
Current Data Parameters
NAME Jan17fw1003C
EXPNO 5
PROCNO 1
F2 - Acquisition Parameters
Date_ 20140117
Time 14.43
INSTRUM spect
PROBHD 5 mm PABBO BB-
PULPROG zgpg30
TD 65536
SOLVENT MeOD
NS 300
DS 4
SWH 24038.461 Hz
FIDRES 0.366798 Hz
AQ 1.3631488 sec
RG 194.02
DW 20.800 usec
DE 6.50 usec
TE 299.7 K
D1 1.50000000 sec
D11 0.03000000 sec
TD0 1
======= CHANNEL f1 =======
SFO1 100.6238364 MHz
NUC1 13C
P1 10.00 usec
PLW1 60.00000000 W
======= CHANNEL f2 =======
SFO2 400.1316005 MHz
NUC2 1H
CPDPRG[2 waltz16
PCPD2 80.00 usec
PLW2 17.20000076 W
PLW12 0.47896999 W
PLW13 0.30654001 W
F2 - Processing parameters
SI 32768
SF 100.6126287 MHz
WDW EM
SSB 0
LB 1.00 Hz
GB 0
PC 1.40

Compound 3 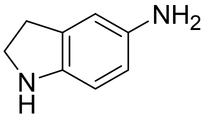
NH2
N
H

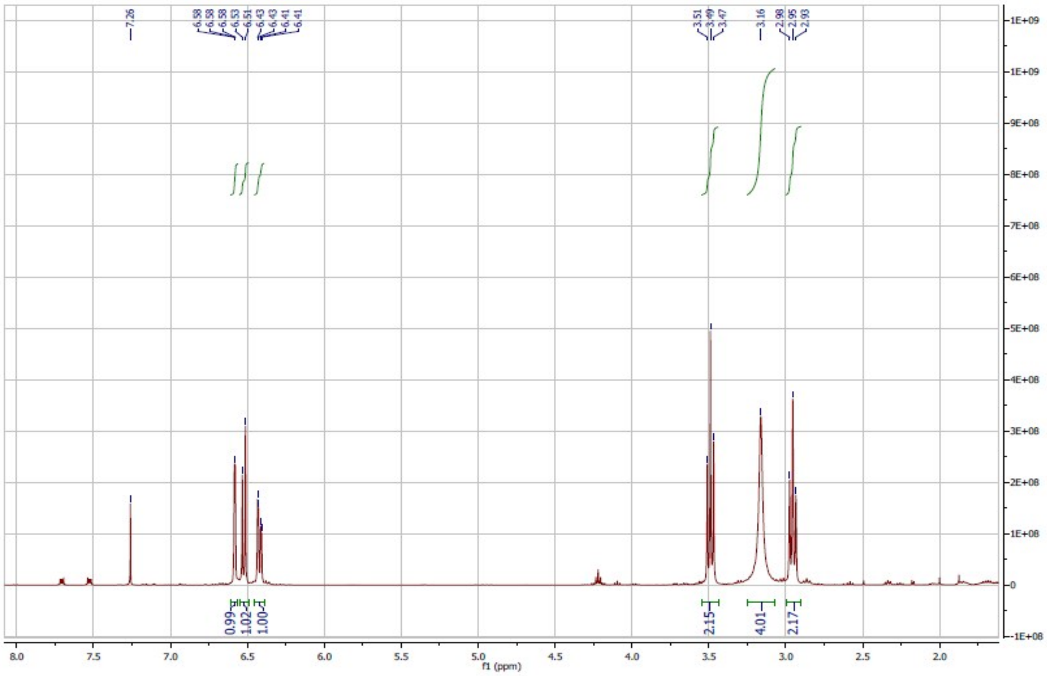
7.26
6.58
6.58
6.58
6.53
6.51
6.43
6.43
6.41
6.41
3.51
3.49
3.47
3.16
2.98
2.95
2.93
0.99
1.02
1.00
2.15
4.01
2.17
f1 (ppm)

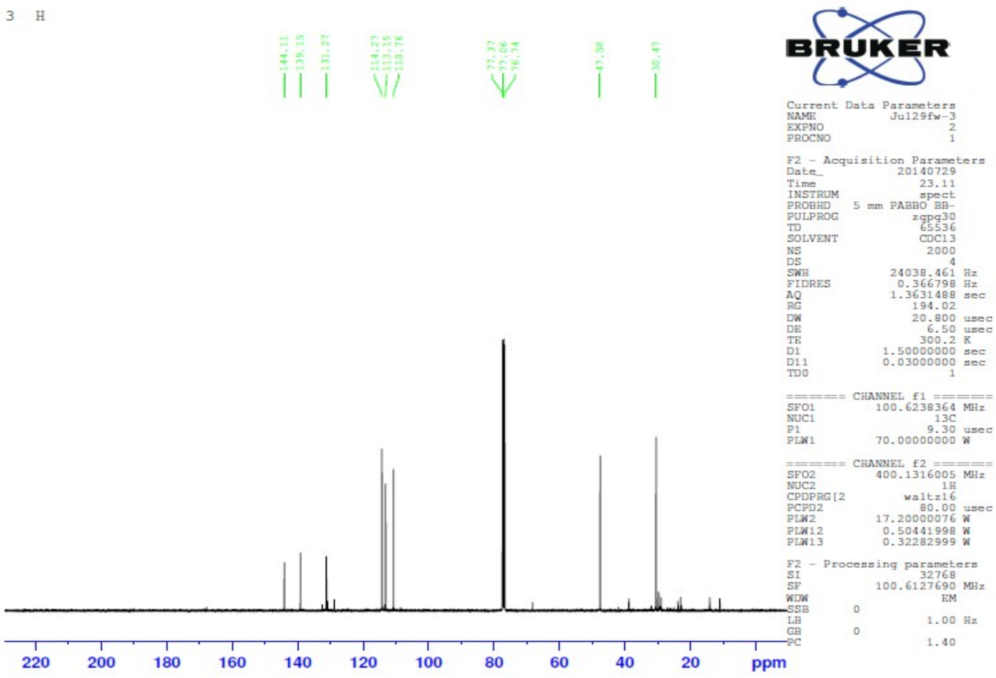
3 H
144.11
139.15
131.27
114.27
113.15
110.76
77.37
77.06
76.74
47.58
30.47
BRUKER
Current Data Parameters
NAME Jul29fw-3
EXPNO 2
PROCNO 1
F2 - Acquisition Parameters
Date_ 20140729
Time 23.11
INSTRUM spect
PROBHD 5 mm PABBO BB-
PULPROG zgpg30
TD 65536
SOLVENT CDCl3
NS 2000
DS 4
SWH 24038.461 Hz
FIDRES 0.366798 Hz
AQ 1.3631488 sec
RG 194.02
DW 20.800 usec
DE 6.50 usec
TE 300.2 K
D1 1.50000000 sec
D11 0.03000000 sec
TD0 1
======= CHANNEL f1 =======
SFO1 100.6238364 MHz
NUC1 13C
P1 9.30 usec
PLW1 70.00000000 W
======= CHANNEL f2 =======
SFO2 400.1316005 MHz
NUC2 1H
CPDPRG[2 waltz16
PCPD2 80.00 usec
PLW2 17.20000076 W
PLW12 0.50441998 W
PLW13 0.32282999 W
F2 - Processing parameters
SI 32768
SF 100.6127690 MHz
WDW EM
SSB 0
LB 1.00 Hz
GB 0
PC 1.40
ppm

Compound 4 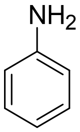
NH2

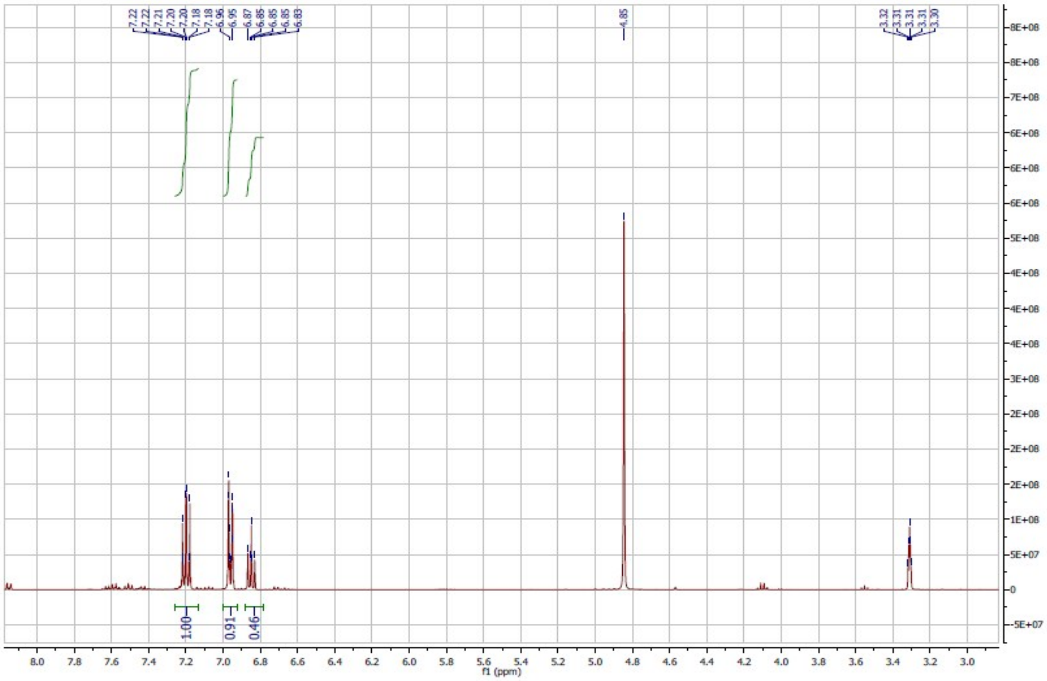
7.22
7.22
7.21
7.20
7.20
7.18
7.18
6.96
6.95
6.87
6.85
6.85
6.85
6.85
6.83
4.85
3.32
3.31
3.31
3.31
3.30
1.00
0.91
0.46
f1 (ppm)

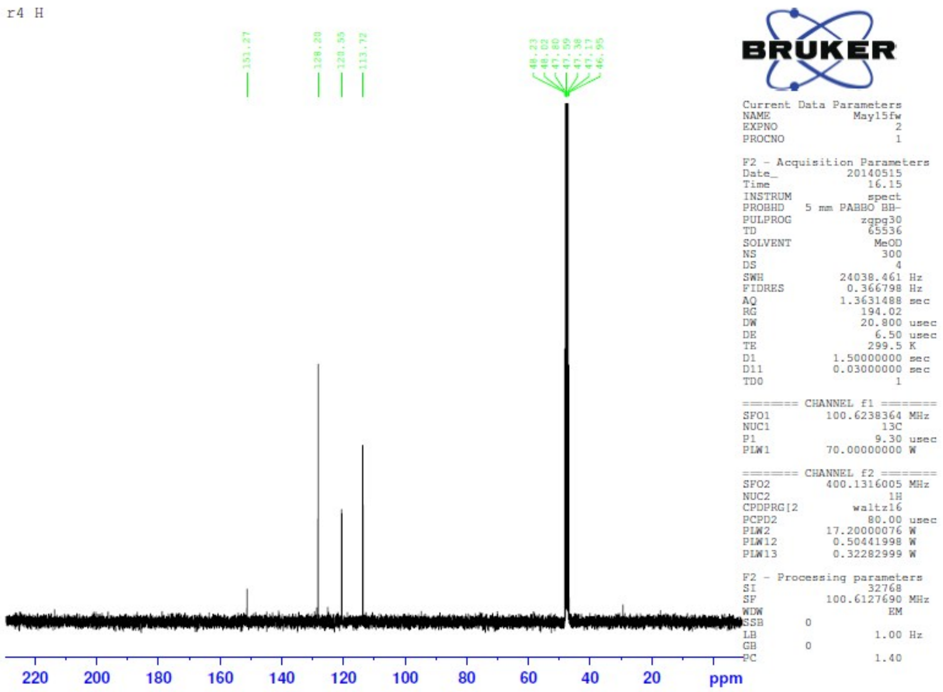
r4 H
151.27
128.28
120.55
113.72
48.23
48.02
47.80
47.59
47.38
47.17
46.95
BRUKER
Current Data Parameters
NAME May15fw
EXPNO 2
PROCNO 1
F2 - Acquisition Parameters
Date_ 20140515
Time 16.15
INSTRUM spect
PROBHD 5 mm PABBO BB-
PULPROG zgpg30
TD 65536
SOLVENT MeOD
NS 300
DS 4
SWH 24038.461 Hz
FIDRES 0.366798 Hz
AQ 1.3631488 sec
RG 194.02
DW 20.800 usec
DE 6.50 usec
TE 299.5 K
D1 1.50000000 sec
D11 0.03000000 sec
TD0 1
======= CHANNEL f1 =======
SFO1 100.6238364 MHz
NUC1 13C
P1 9.30 usec
PLW1 70.00000000 W
======= CHANNEL f2 =======
SFO2 400.1316005 MHz
NUC2 1H
CPDPRG[2 waltz16
PCPD2 80.00 usec
PLW2 17.20000076 W
PLW12 0.50441998 W
PLW13 0.32282999 W
F2 - Processing parameters
SI 32768
SF 100.6127690 MHz
WDW EM
SSB 0
LB 1.00 Hz
GB 0
PC 1.40
ppm

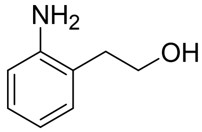

NH2
OH

Compound 5

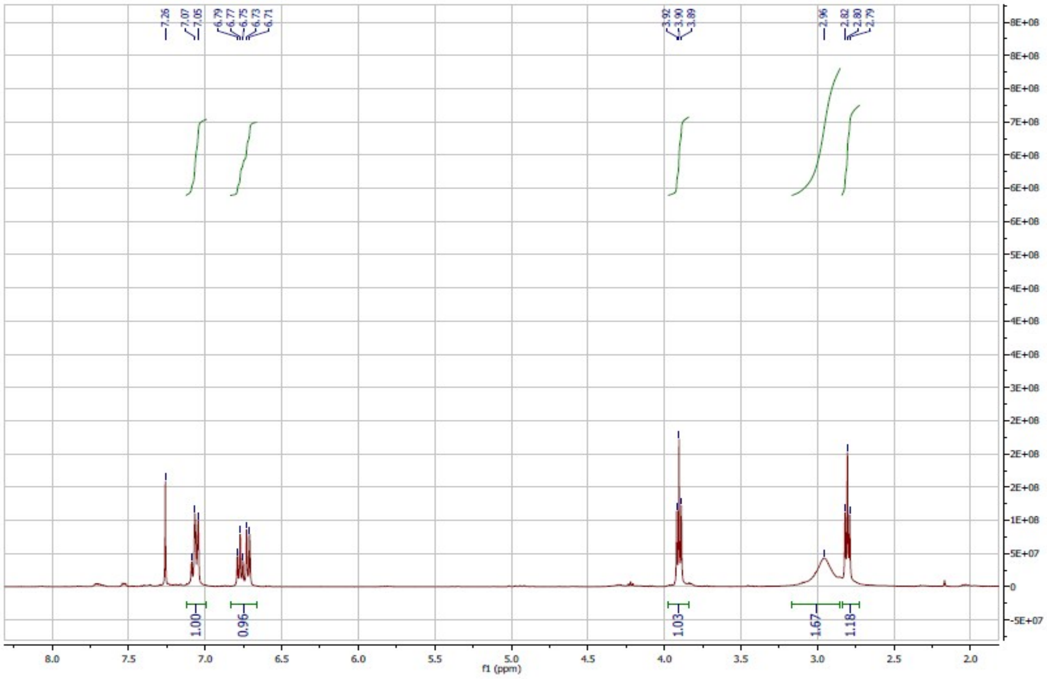

7.26
7.07
7.05
6.79
6.77
6.75
6.73
6.71
3.92
3.90
3.89
2.96
2.82
2.80
2.79
1.00
0.96
1.03
1.67
1.18
f1 (ppm)

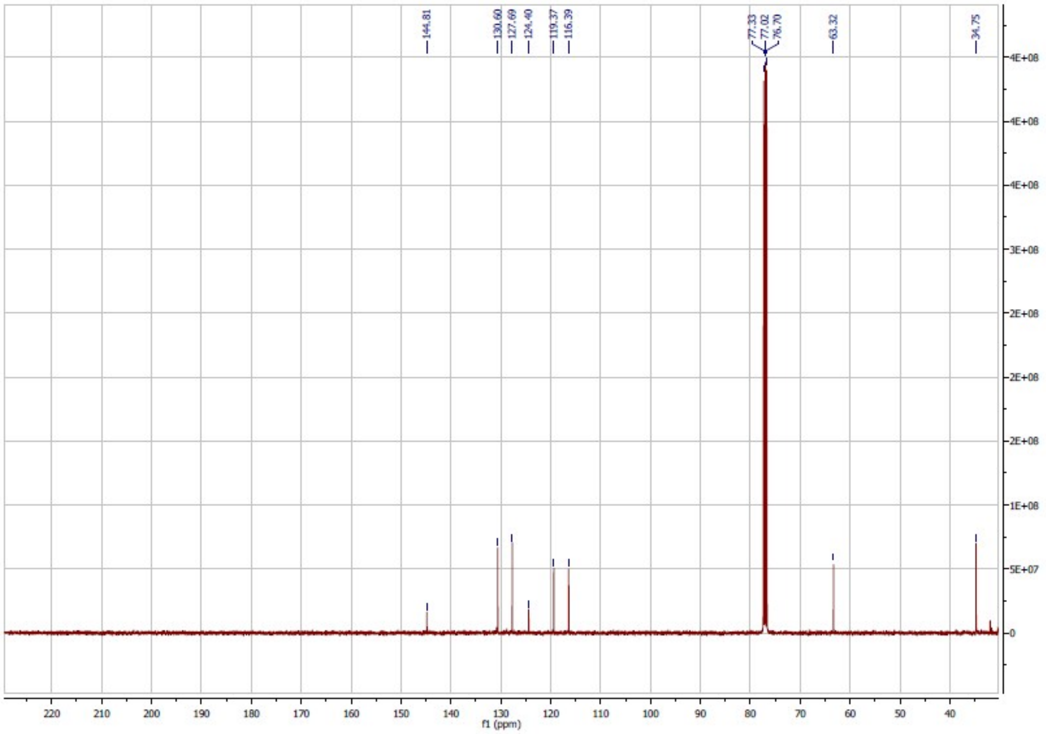

144.81
130.60
127.69
124.40
119.37
116.39
77.33
77.02
76.70
63.32
34.75
f1 (ppm)

Compound 6

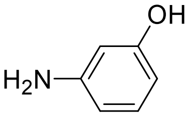

OH
H2N

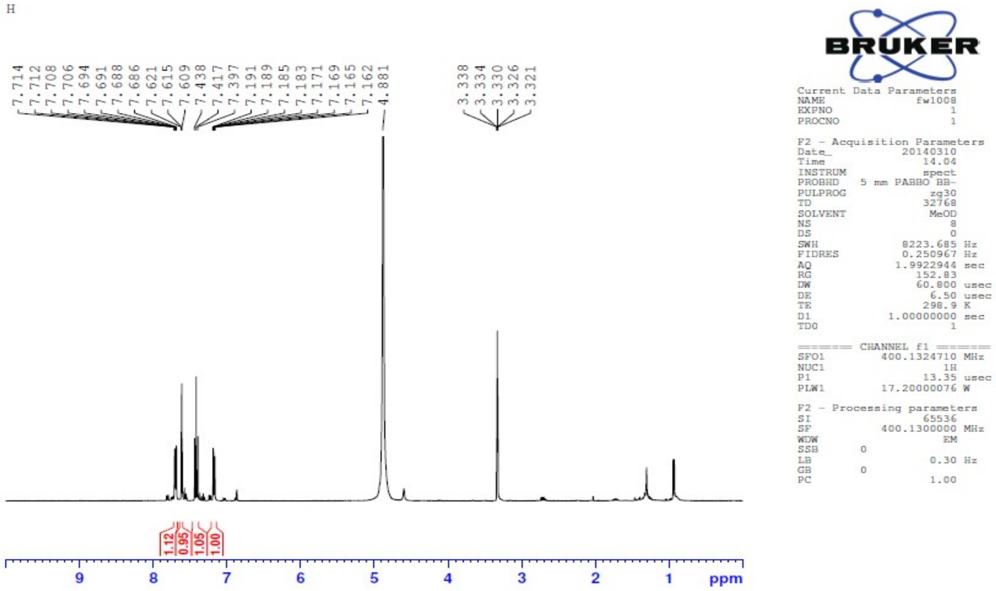

H
7.714
7.712
7.708
7.706
7.694
7.691
7.688
7.686
7.621
7.615
7.609
7.438
7.417
7.397
7.191
7.189
7.185
7.183
7.171
7.169
7.165
7.162
4.881
3.338
3.334
3.330
3.326
3.321
1.12
0.95
1.05
1.00
9
8
7
6
5
4
3
2
1
ppm
BRUKER
Current Data Parameters
NAME fw1008
EXPNO 1
PROCNO 1
F2 - Acquisition Parameters
Date_ 20140310
Time 14.04
INSTRUM spect
PROBHD 5 mm PABBO BB-
PULPROG zg30
TD 32768
SOLVENT MeOD
NS 8
DS 0
SWH 8223.685 Hz
FIDRES 0.250967 Hz
AQ 1.9922944 sec
RG 152.83
DW 60.800 usec
DE 6.50 usec
TE 298.9 K
D1 1.00000000 sec
TD0 1
======= CHANNEL f1 =======
SFO1 400.1324710 MHz
NUC1 1H
P1 13.35 usec
PLW1 17.20000076 W
F2 - Processing parameters
SI 65536
SF 400.1300000 MHz
WDW EM
SSB 0
LB 0.30 Hz
GB 0
PC 1.00

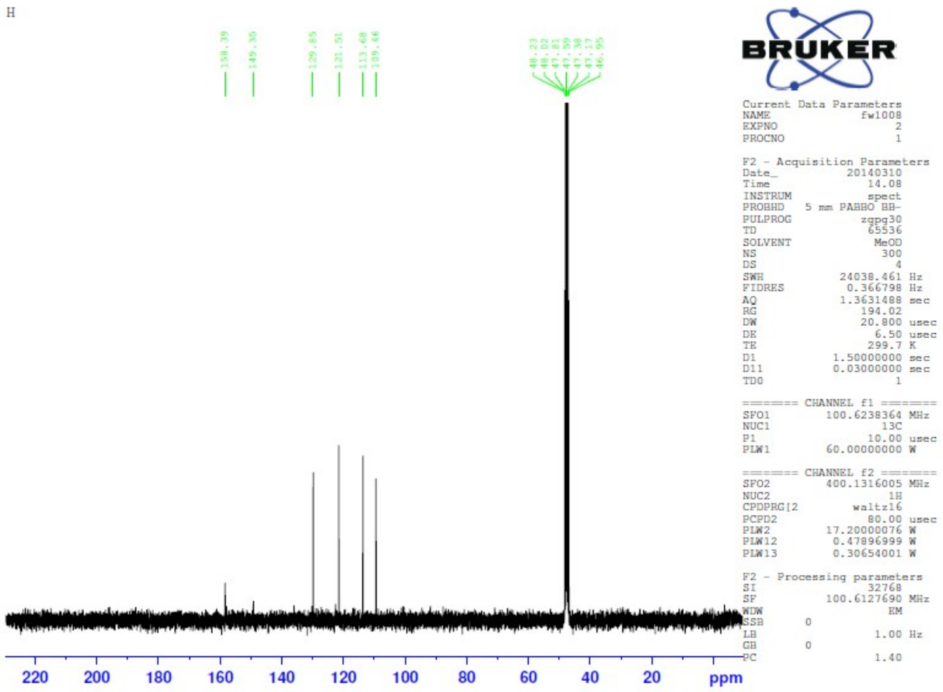

H
158.39
149.35
129.85
121.51
113.68
109.46
48.23
48.02
47.81
47.59
47.38
47.17
46.95
220
200
180
160
140
120
100
80
60
40
20
ppm
BRUKER
Current Data Parameters
NAME fw1008
EXPNO 2
PROCNO 1
F2 - Acquisition Parameters
Date_ 20140310
Time 14.08
INSTRUM spect
PROBHD 5 mm PABBO BB-
PULPROG zgpg30
TD 65536
SOLVENT MeOD
NS 300
DS 4
SWH 24038.461 Hz
FIDRES 0.366798 Hz
AQ 1.3631488 sec
RG 194.02
DW 20.800 usec
DE 6.50 usec
TE 299.7 K
D1 1.50000000 sec
D11 0.03000000 sec
TD0 1
======= CHANNEL f1 =======
SFO1 100.6238364 MHz
NUC1 13C
P1 10.00 usec
PLW1 60.00000000 W
======= CHANNEL f2 =======
SFO2 400.1316005 MHz
NUC2 1H
CPDPRG[2 waltz16
PCPD2 80.00 usec
PLW2 17.20000076 W
PLW12 0.47896999 W
PLW13 0.30654001 W
F2 - Processing parameters
SI 32768
SF 100.6127690 MHz
WDW EM
SSB 0
LB 1.00 Hz
GB 0
PC 1.40

Compound 7 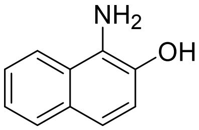
NH2
OH

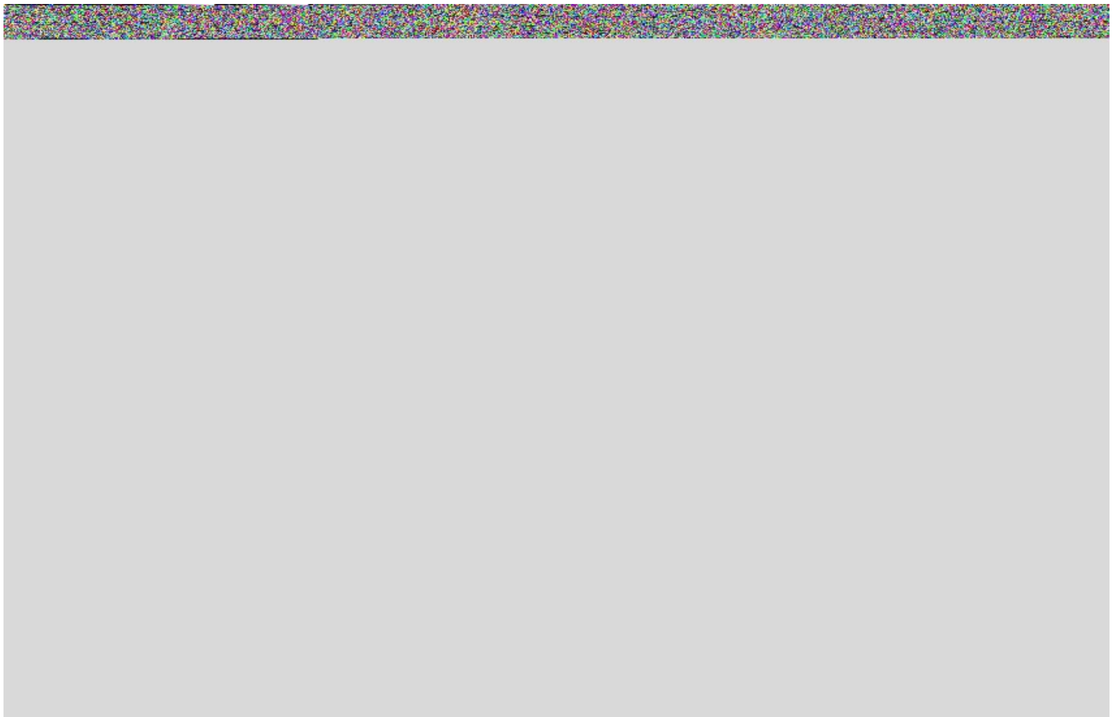

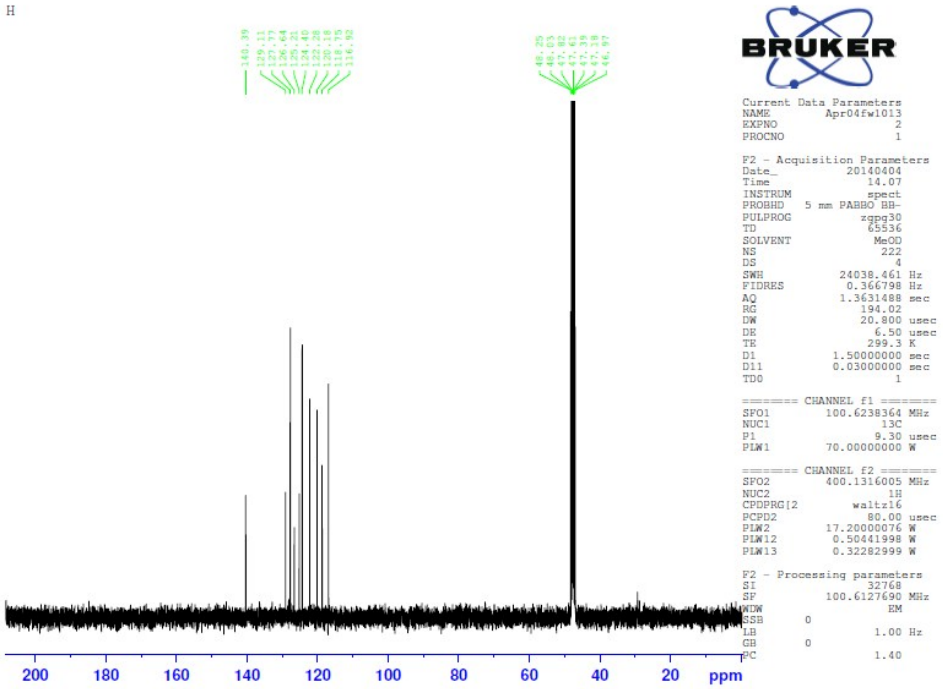
H
140.39
129.11
127.77
126.64
125.21
124.40
122.28
120.18
118.75
116.92
48.25
48.03
47.82
47.61
47.39
47.18
46.97
200
180
160
140
120
100
80
60
40
20
ppm
BRUKER
Current Data Parameters
NAME Apr04fw1013
EXPNO 2
PROCNO 1
F2 - Acquisition Parameters
Date_ 20140404
Time 14.07
INSTRUM spect
PROBHD 5 mm PABBO BB-
PULPROG zgpg30
TD 65536
SOLVENT MeOD
NS 222
DS 4
SWH 24038.461 Hz
FIDRES 0.366798 Hz
AQ 1.3631488 sec
RG 194.02
DW 20.800 usec
DE 6.50 usec
TE 299.3 K
D1 1.50000000 sec
D11 0.03000000 sec
TD0 1
======== CHANNEL f1 ========
SFO1 100.6238364 MHz
NUC1 13C
P1 9.30 usec
PLW1 70.00000000 W
======== CHANNEL f2 ========
SFO2 400.1316005 MHz
NUC2 1H
CPDPRG[2 waltz16
PCPD2 80.00 usec
PLW2 17.20000076 W
PLW12 0.50441998 W
PLW13 0.32282999 W
F2 - Processing parameters
SI 32768
SF 100.6127690 MHz
WDW EM
SSB 0
LB 1.00 Hz
GB 0
PC 1.40

Compound 8

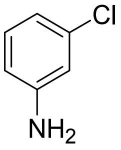
Cl
NH2

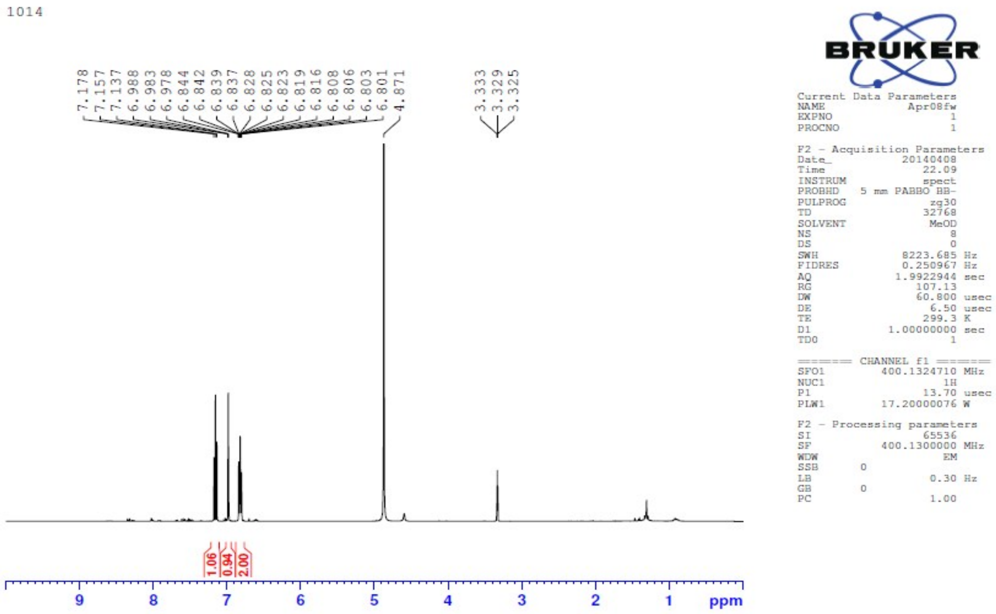
1014
7.178
7.157
7.137
6.988
6.983
6.978
6.844
6.842
6.839
6.837
6.828
6.825
6.823
6.819
6.816
6.808
6.806
6.803
6.801
4.871
3.333
3.329
3.325
1.06
0.94
2.00
9 8 7 6 5 4 3 2 1 ppm
BRUKER
Current Data Parameters
NAME Apr08fw
EXPNO 1
PROCNO 1
F2 - Acquisition Parameters
Date_ 20140408
Time 22.09
INSTRUM spect
PROBHD 5 mm PABBO BB-
PULPROG zg30
TD 32768
SOLVENT MeOD
NS 8
DS 0
SWH 8223.685 Hz
FIDRES 0.250967 Hz
AQ 1.9922944 sec
RG 107.13
DW 60.800 usec
DE 6.50 usec
TE 299.3 K
D1 1.00000000 sec
TD0 1
======= CHANNEL f1 =======
SFO1 400.1324710 MHz
NUC1 1H
P1 13.70 usec
PLW1 17.20000076 W
F2 - Processing parameters
SI 65536
SF 400.1300000 MHz
WDW EM
SSB 0
LB 0.30 Hz
GB 0
PC 1.00

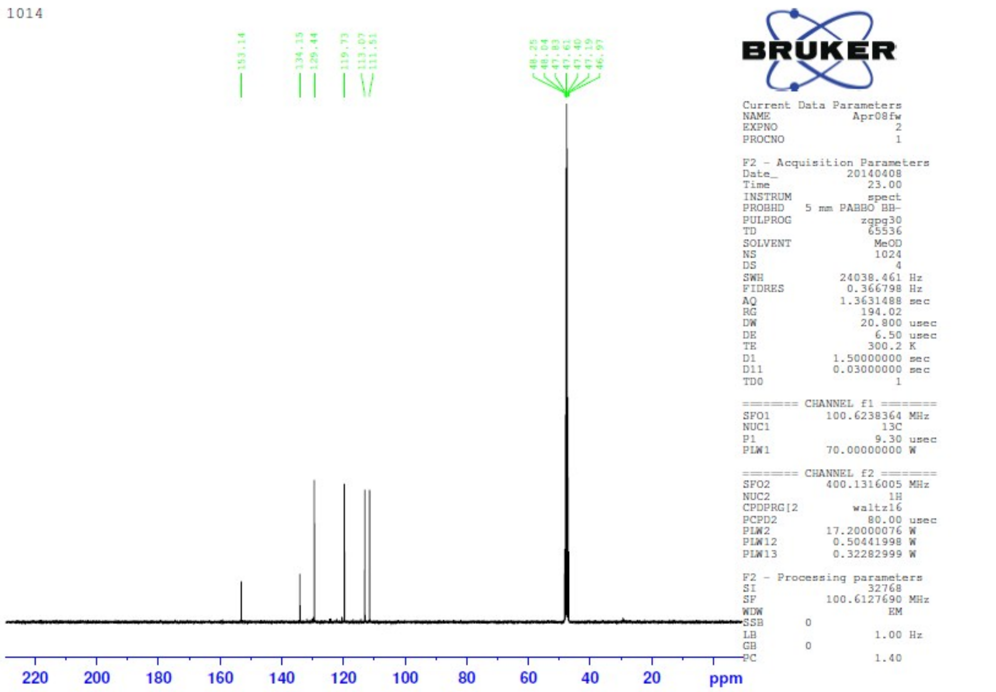
1014
153.14
134.15
129.44
119.73
113.07
111.51
48.25
48.04
47.83
47.61
47.40
47.19
46.97
220 200 180 160 140 120 100 80 60 40 20 ppm
BRUKER
Current Data Parameters
NAME Apr08fw
EXPNO 2
PROCNO 1
F2 - Acquisition Parameters
Date_ 20140408
Time 23.00
INSTRUM spect
PROBHD 5 mm PABBO BB-
PULPROG zgpg30
TD 65536
SOLVENT MeOD
NS 1024
DS 4
SWH 24038.461 Hz
FIDRES 0.366798 Hz
AQ 1.3631488 sec
RG 194.02
DW 20.800 usec
DE 6.50 usec
TE 300.2 K
D1 1.50000000 sec
D11 0.03000000 sec
TD0 1
======= CHANNEL f1 =======
SFO1 100.6238364 MHz
NUC1 13C
P1 9.30 usec
PLW1 70.00000000 W
======= CHANNEL f2 =======
SFO2 400.1316005 MHz
NUC2 1H
CPDPRG[2 waltz16
PCPD2 80.00 usec
PLW2 17.20000076 W
PLW12 0.50441998 W
PLW13 0.32282999 W
F2 - Processing parameters
SI 32768
SF 100.6127690 MHz
WDW EM
SSB 0
LB 1.00 Hz
GB 0
PC 1.40

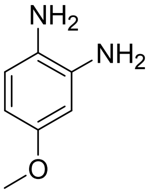
NH2
NH2
O

Compound 9

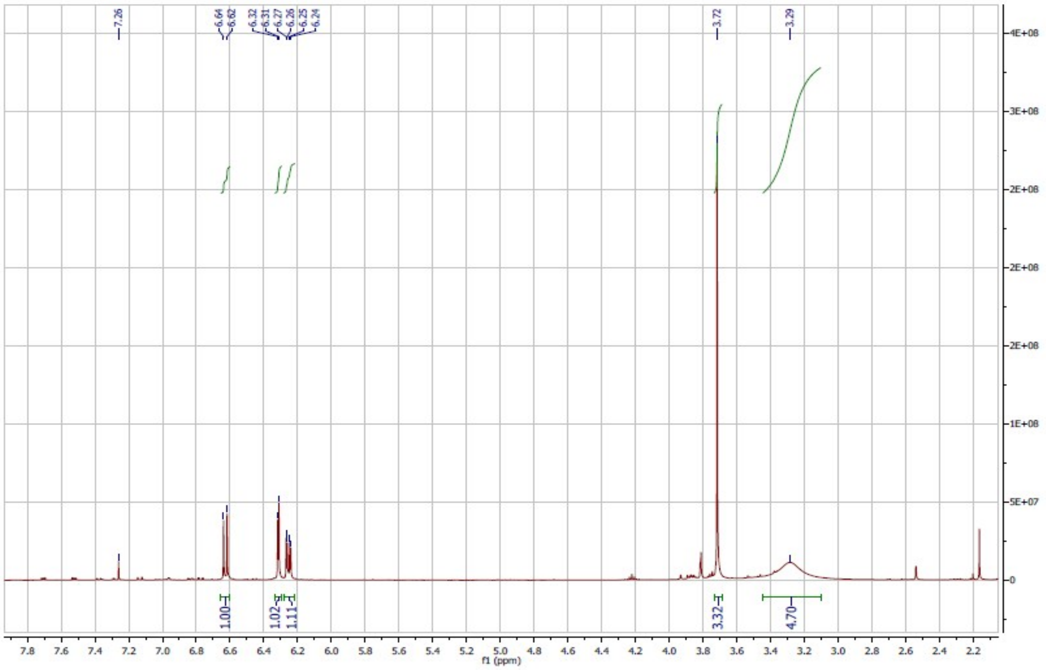
7.26
6.64
6.62
6.32
6.31
6.27
6.26
6.25
6.24
3.72
3.29
1.00
1.02
1.11
3.32
4.70
f1 (ppm)

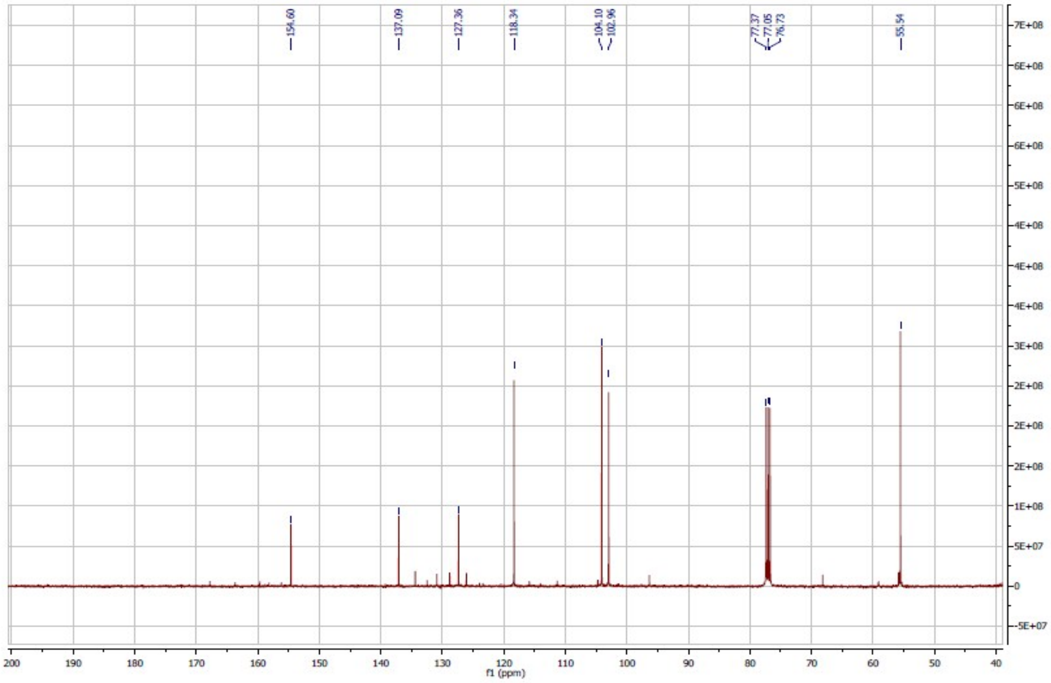
154.60
137.09
127.36
118.34
104.10
102.96
77.37
77.05
76.73
55.54
f1 (ppm)
